# Species Composition, Growth, and Trophic Traits of Hairtail (Trichiuridae), the Most Productive Fish in Chinese Marine Fishery

**DOI:** 10.3390/ani12223078

**Published:** 2022-11-08

**Authors:** Xiongbo He, Zhisen Luo, Chunxu Zhao, Liangliang Huang, Yunrong Yan, Bin Kang

**Affiliations:** 1 Fisheries College, Guangdong Ocean University, Zhanjiang 524088, China; 2 Guangdong Provincial Engineering and Technology Research Center of Far Sea Fisheries Management and Fishing of South China Sea, Guangdong Ocean University, Zhanjiang 524088, China; 3 Southern Marine Science and Engineering Guangdong Laboratory (Zhanjiang), Zhanjiang 524000, China; 4 College of Environmental Science and Engineering, Guilin University of Technology, Guilin 541006, China; 5 Fisheries College, Ocean University of China, Qingdao 266003, China

**Keywords:** taxonomy, distribution, otolith, stable isotope, trophic level

## Abstract

**Simple Summary:**

This study aims to map the species composition of catches and the distribution of hairtail in Chinese seas, and to determine the growth, age, and trophic characteristics of different species. The results of this work indicated that six hairtail species were collected and most individuals were at ages of 1–2 years; largehead hairtail showed the highest stability and the greatest impact on the stability of the trophic structure.

**Abstract:**

Hairtail (Scombriformes: Trichiuridae) have always ranked first in single-species production in Chinese marine fishery. However, due to the confusion of species identification, in official statistics, data on hairtail production and economic value are a combination of all the different species of Trichiuridae. In this study, based on sampling along China’s coastal areas, the composition and distribution of common hairtail species, as well as their age, growth, and trophic traits, are discussed. Six species of four genera and two subfamilies were identified, and largehead hairtail (*Trichiurus japonicus*) and Savalai hairtail (*Lepturacanthus savala*) were the most important populations that constituted catch production. The growth parameter *b* could be divided into two groups, with values in species mostly inhabiting northern parts of Chinese seas higher than those in southern parts. Most individuals were 1–2 years of age, suggesting species sexual precocity and individual miniaturization under multiple stresses. Species of Trichiuridae are at the top of the food web and play a bottom-up role in regulating the trophic dynamics of marine food webs. Largehead hairtail showed the highest stability and the greatest impact on the stability of the trophic structure. Despite temporary resource maintenance under fishery-induced evolution, the effective recovery and reasonable utilization of hairtail resources are still the main focuses of attention for Chinese marine fishery.

## 1. Introduction

Globally, marine fisheries have generally declined, and species compositions have undergone great changes [1,2]. Hairtail (Scombriformes: Trichiuridae), mainly distributed in the Northwest Pacific, Indian, and Atlantic Oceans [3], are one of the main fishing objects in global and Chinese fishery, with an average annual production of 1.31 million tons from 2005 to 2016 in the world, with about 80% coming from China [2,4]. Under the pressure of high-intensity fishing in China, the production of traditional economic fishes, such as wild large yellow croaker and small yellow croaker, has sharply declined [5,6]. However, the output of hairtail has always maintained a high yield, ranking first in single-species production in Chinese marine fishery [4].

Stark [7] put forward the view that the hairtail family may have evolved from Ophiopovidae through a comparative study of the skeletal system. Before the 1960s, there was only one species of hairtail accepted all over the world, after which more and more new species of Trichiuridae were discovered as a benefit of strengthening sampling efforts and the development of taxonomic knowledge. Currently, 47 species in 10 genera of Trichiuridae are recognized all over the world [8], among which 11 species of 8 genera were reported in China [9]. As migratory species covering a wide range of habitats with different food sources and environmental conditions, species compositions in different areas are different, and the same species may present different populations along the China coastline [10]. Possibly due to a lack of sufficient knowledge on hairtail taxonomy or no price difference among different species, some fishermen group hairtail in their catches; thus, in official statistics, data on hairtail production and economic value are a combination of all the different species of Trichiuridae. This has led to the problem of species compositions and population structures of hairtail in China still being unclear.

In addition to variations in production and catch composition, fishery change is also reflected in species growth, through the relationship between body length and weight, which can be expressed by a power function. In FishBase, parameters for the length-weight relationship of many fishes are recorded [3], providing a favorable reference for the assessment of world fishery, e.g., the allometric growth parameter *b* related to different growth stages and nutritional conditions can be used to judge growth pattern and reflect the status quo of population structure to a certain extent [11]. Values of parameter *b* generally fall within the range of 2.0–4.0, of which 95% fall within the normal range of 2.5–3.5; when *b* equals 3, a fish body shows uniform growth, while less than 3 or more than 3 are, respectively, negative and positive allometric growth [12].

The interspecific trophic niche relationship is also a major indicator of fishery change. According to the principle that the stable isotope ratio of a consumer is close to the corresponding isotope ratio of its food, carbon and nitrogen isotope ratios are used to express the multi-dimensional ecological space information of species, including carbon for determining food source and nitrogen for determining trophic level [13,14]. Borrowing from the morphological method, six trophic ecological indicators, including range of δ^13^C (CR), range of δ^15^N (NR), total area of the convex hull (TA), mean distance to centroid (CD), mean nearest neighbor distance (MNND), and standard deviation of nearest neighbor distance (SDNND), were further proposed based on a carbon and nitrogen stable isotope double bitmap to quantitatively analyze species’ trophic structures [15]. Based on these six indicators, standard elliptical area (SEA) was further proposed as a more accurate evaluation indicator of core niche width by excluding the impact of deviation values [16].

Based on a large-scale survey of hairtail along Chinese coastal areas, in this study, the species compositions of catches and their distributions are mapped, and the growth, age, and trophic characteristics of different species are determined. The results are evaluated to propose and implement more effective development and protection strategies for hairtail resources.

## 2. Materials and Methods

### 2.1. Specimen Collection

In the autumn of 2018, just after the fishing moratorium, Trichiuridae specimens were collected at 17 fishing ports along the coast of China, and the geographical information of the sampling sites and sampling nets was acquired from the fishermen (Table 1). A total of 2281 individuals were collected, mostly from trawl catches, purse seines, and gill nets.

### 2.2. Species Morphometry and Identification

In total, 17 morphological characteristics (Appendix A) were measured and compared to identify species, according to the “Chinese marine fish records” [9] and FishBase [3], and then revised using the Catalog of Fishes [8] to remove synonyms and homonyms.

### 2.3. Age Determination

All the specimens were grouped into different preanal length groups (20 mm distance between groups). Intact left otoliths of three individuals of each group were randomly sampled from each species for age determination, and a total of 292 otoliths were obtained. Otoliths were cleaned to remove tissue film and impurities on the surface and then individually embedded in a custom mold using a specific ratio of reagents (acrylic resin powder: curing agent, 0.8:1) [17]. Embedded samples were ground using sandpaper with specifications of 240 mesh, 600 mesh, 1200 mesh, and 2000 mesh in turn until the otolith core and the age pattern could be clearly examined under a microscope. Finally, the ground surfaces of the otoliths were polished with cloth and aluminite powder at 0.3 μm [17,18].

The otolith slices were observed under an optical microscope (Olympus BX51) equipped with a camera device (ARTCAM-130MI, Artray Co., Ltd., Tokyo, Japan) [19]. The opaque zone that spread out from the core of the otolith was designated as the annual ring, and its combination with the transparent zone next to the inner side represented 1 years of age (Appendix B). The annual rings were counted for each sample. To avoid personal error, at least two coherent reads out of three were needed to confirm the fish age value [17,18,19].

### 2.4. Stable Isotope Measurement

An appropriate amount of muscle was sampled from the middle of the back of each individual. The samples were stored in a freeze dryer (ALPHA1-2LD plus lyophilization machines) for dehydration treatment at a temperature of −48 °C for 48 h. The dried muscles were ground into fine powder for stable isotope measurement using a grinding tool (BIOSPEC MiniBeadbeater-16). Stable carbon and nitrogen isotopes were measured with an elemental analyzer (Carlo Erba EA-1110) and a continuous-flow isotope ratio mass spectrometer (Delta Plus Finnigan). A standard sample was inserted every 10 samples, and 1–2 samples were randomly selected for repeated testing to ensure measurement accuracy [20,21].

### 2.5. Data Analysis

To exclude the influence of individual size on morphological characteristics, the original measurement data were divided by preanal length to obtain the characteristic parameter ratio. Morphological parameters were grouped by principal component analysis, and the load coefficient and contribution rate of each principal component were determined.

The relationship between species preanal length and body weight was described as follows:

*W* = *a L ^b^*

where *L* is the of preanal length (mm), *W* is body weight (g), *a* is the conditional parameter, and *b* is the allometric growth parameter [12].

The relationship between preanal length and the radius of the otolith section was fitted using an exponential function:

$$L=a_{1}e^{Rb_{1}}$$

where *R* is the radius of the otolith section, and *a*_1_ and *b*_1_ are undetermined parameters [19].

The relationship between preanal length and age was fitted using the von Bertalanffy growth equation:

*L*_t_ = *L*_∞_ (1 − e^−*k* (*t* − *t*0)^)

where *L*_t_ is the preanal length of an individual at age *t*, *L_∞_* is the limit of preanal length, *K* is the growth parameter, and *t*_0_ is the theoretical preanal length at age 0 [22].

The ratios of carbon isotopes and nitrogen isotopes were represented by δ^13^C and δ^15^N, respectively, and could be determined as follows [23]:

δ*X* (‰) = [(*R*_sample_/*R*_standard_) − 1] × 1000

where *X* is ^13^C or ^15^N, and *R* is ^13^C/^12^C or ^15^N/^14^N. Vienna Pee Dee Belemnite (VPDB) [23,24] and the Earth’s atmospheric N_2_ [25,26] were used as standards for C and N, respectively.

Species trophic level was determined by the differences among nitrogen stable isotopes, baseline organisms, and fractionation:

TL = (δ^15^N_sample_ − δ^15^N_0_)/δ^15^N_c_ + TL_b_

where TL is the species trophic level, δ^15^N_sample_ is the nitrogen stable isotope value of the fish sample, δ^15^N_0_ is the nitrogen stable isotope value of the baseline organism, δ^15^N_c_ is the trophic level of enrichment, and TL_b_ is the trophic level of the baseline organism. In this study, local common shellfish species were selected as baseline organisms [21]. The trophic level enrichment was taken as 3.4 ‰, and the trophic level of baseline organisms was taken as 2 [13].

The stable isotope mixed model of SIAR (stable isotope analysis in R) was used to draw a convex polygon enclosed by a two-dimensional point set, as well as a standard ellipse according to the core of data distribution, based on which trophic indicators of CR, NR, TA, CD, MNND, SDNND, and SEA were determined [15,16].

## 3. Results

### 3.1. Common Species Composition of Trichiuridae in Chinese Seas

According to differences in morphological characteristics, such as morphometric characteristics including the ratio of body full length to preanal length, countable characteristics including the number of fin rays and number of gill rakers, description characteristics including downward curvature of the lateral line above the pectoral fin, and development of the first anal spine, six species of four genera and two subfamilies were identified, including South China Sea hairtail (*Trichiurus nanhaiensis* Wang & Xu, 1992), Chinese short-tailed hairtail (*Trichiurus brevis* Wang & You, 1992), and largehead hairtail (*Trichiurus japonicus* Temminck & Schlegel, 1844) of genus *Trichiurus*; crested hairtail *Tentoriceps cristatus* (Klunzinger, 1884) of the genus *Tentoriceps*; Savalai hairtail (*Lepturacanthus savala* (Cuvier, 1829)) of the genus *Lepturacanthus* in the subfamily Trichiurinae; and smallhead hairtail (*Eupleurogrammus muticus* (Gray, 1831)) of the genus *Eupleurogrammus* in the subfamily Lepidopodinae (Appendix C).

**Figure 1:**
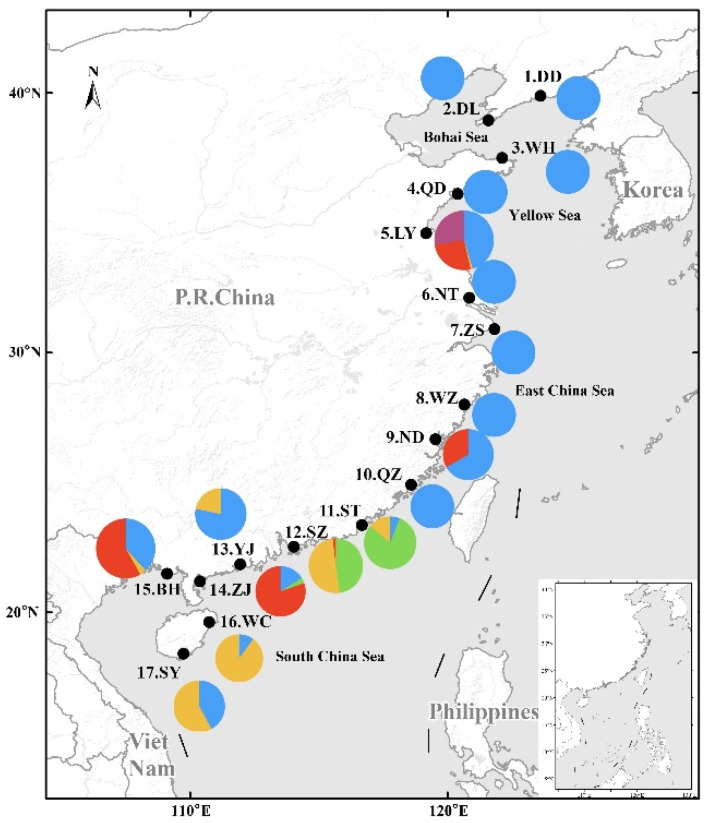
Composition and distribution of six species of Trichiuridae in China’s coastal waters. The abbreviation of sampling sites name was the same of Table 1. Different colors represented different species: blue, *Trichiurus japonicus*; green, *T. nanhaiensis*; yellow, *T. brevis*; red, *Lepturacanthus savala*; purple, *Eupleurogrammus muticus*; light blue, *Tentoriceps cristatus*.

Among 2281 individuals, there were 1340 largehead hairtail, accounting for 58.75% of the total samples. Largehead hairtail were widely distributed at 16 of the 17 sampling sites, followed by Chinese short-tailed hairtail at eight sites, Savalai hairtail at six sites, South China Sea hairtail at five sites, and smallhead hairtail and crested hairtail at two sites each. Hairtail species showed obvious differences in spatial distribution from north to south: there were only largehead hairtail in the Bohai and Yellow seas (except Lianyungang with four species) and the East China Sea (except Ningde, with two species); in the South China Sea, all six species were sampled, especially in Shantou, with five species (all species except smallhead hairtail) (Figure 1).

### 3.2. Species Morphometry

The six species of hairtail showed a wide range of preanal lengths and body weights (Table 2).

**Figure 2:**
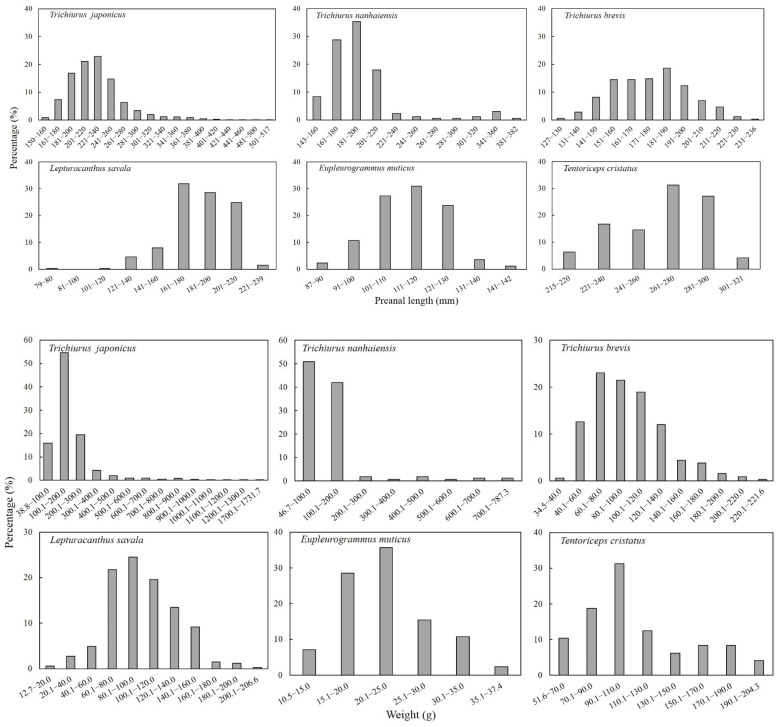
Population composition in terms of preanal length and body weight of six species of Trichiuridae in Chinese seas.

For largehead hairtail, the dominant preanal length and the dominant body weight were 181–260 mm and 0.1–300.0 g, respectively, accounting for 76% and 90% of the samples of this species. For South China Sea hairtail, the dominant preanal length and body weight were 161–220 mm and 46.8–200.0 g, respectively, accounting for 81% and 93% of the samples of this species. For Chinese short-tailed hairtail, the dominant preanal length was 151–200 mm in 75% of the samples of this species, and the dominant body weight was 40.1–140.0 g in 88.3% of the samples of this species. For Savalai hairtail, the dominant preanal length and body weight were 161–220 mm and 60.1–140.0 g, respectively, accounting for 85% and 79% of the samples of this species. In smallhead hairtail, the dominant preanal length was 101–130 mm and the dominant body weight was 15.1–30.0 g, accounting for 82% and 80% of the samples of this species. For crested hairtail, the dominant preanal length and body weight were 221–300 mm and 51.6–204.2 g, respectively, accounting for 90% and 73% of the samples of this species (Figure 2).

**Figure 3:**
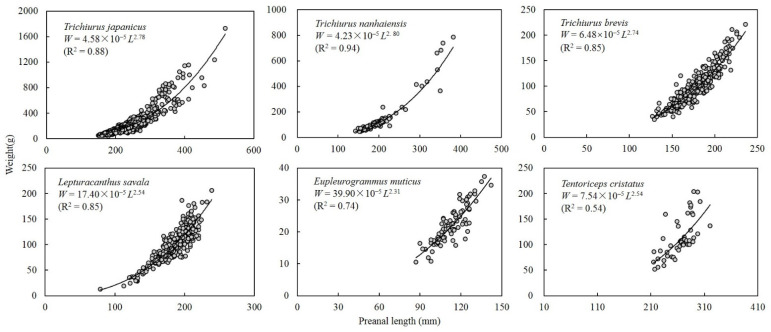
Relationship between preanal length and body weight in six species of Trichiuridae in Chinese seas.

A power function formula was used to describe the relationship between preanal length and body weight (Figure 3). The conditional parameter *a* in smallhead hairtail was significantly higher than that in Savalai hairtail, and both were significantly higher than the other four species, which had no differences. The growth parameter *b* could be divided into two groups, with values in largehead hairtail, South China Sea hairtail, and Chinese short-tailed hairtail higher than those of the other three species.

### 3.3. Age and Growth

In total, 292 otolith samples including 190 largehead hairtail, 43 Chinese short-tailed hairtail, 32 Savalai hairtail, and 27 South China Sea hairtail (smallhead hairtail and crested hairtail with narrow range of preanal length were excluded) were examined to determine individual age (Appendix B). The ages of largehead hairtail had a range of 1–6 years, with the dominant group of 1–2 years accounting for 84% of the total samples, followed by 3 years (12%), 4 years (3%), and 5–6 years (1%); the minimum age of sexual maturity was 2 years. The ages of the other three species of hairtail had ranges of 1–3 years, all with the dominant age of 1 year.

**Figure 4:**
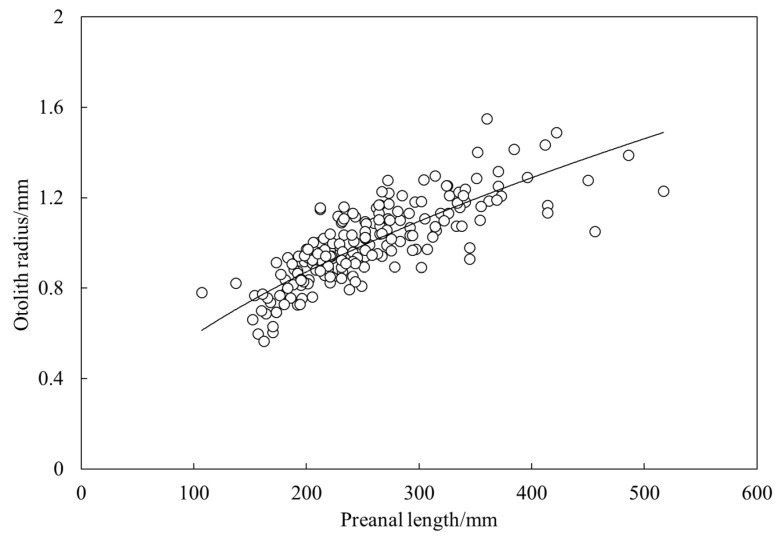
Relationship between preanal lengths and otolith radius of largehead hairtail *Trichiurus japonicus*.

The relationship between preanal length and otolith diameter of largehead hairtail was fitted using a power function as *L* = 0.0442 r^0.563^ (R^2^ = 0.65, *n* = 190) (Figure 4). According to Walford’s growth transformation method, the von Bertalanffy growth equation was *L*_t_ = 666 (1 − e^−0.15 (*t* + 1.19)^) for preanal length and *W*_t_ = 2590 (1 − e^−0.22 (*t* + 0.38)^)^3^ for body weight.

### 3.4. Trophic Structure and Trophic Level

**Figure 5:**
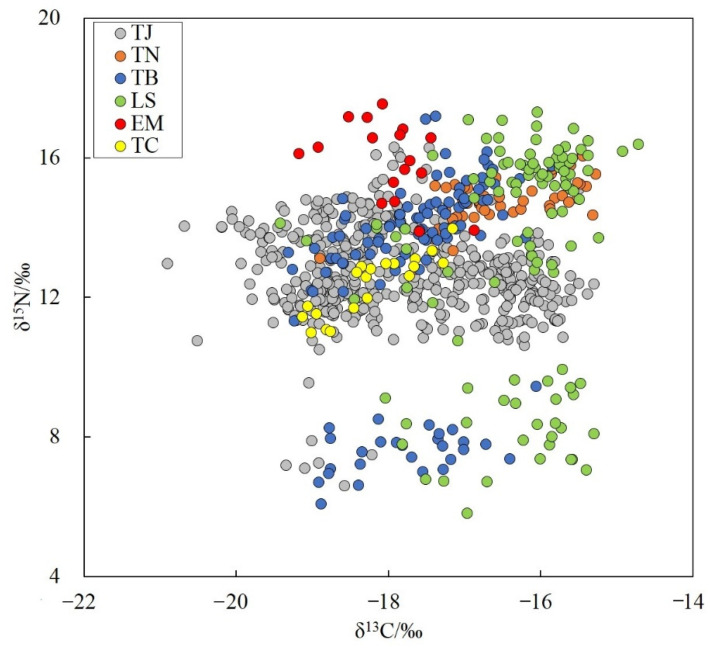
Stable carbon and nitrogen isotopic characteristics of six species of Trichiuridae in Chinese seas. TJ, *Trichiurus japonicus*; TN, *T. nanhaiensis*; TB, *T. brevis*; LS, *Lepturacanthus savala*; EM, *Eupleurogrammus muticus*; TC, *Tentoriceps cristatus*.

The six hairtail species showed wide ranges of carbon and nitrogen stable isotope values, with δ^13^C values from −20.90 to −14.72 ‰, and δ^15^N values from 5.81 to 17.55 ‰, varying among different species. Largehead hairtail showed the largest range of δ^13^C variation of 5.61%, significantly higher than those of other species. For δ^15^N, Savalai hairtail showed the largest range of variation of 11.50%, followed by Chinese short-tailed hairtail at 11.11%, largehead hairtail at 9.71%, smallhead hairtail at 3.66%, and South China Sea hairtail and crested hairtail at 3.32% each (Figure 5). The trophic levels of the six hairtail species were calculated based on nitrogen stable isotopes, ranging from 3.17 for South China Sea hairtail to 3.97 for largehead hairtail.

**Figure 6:**
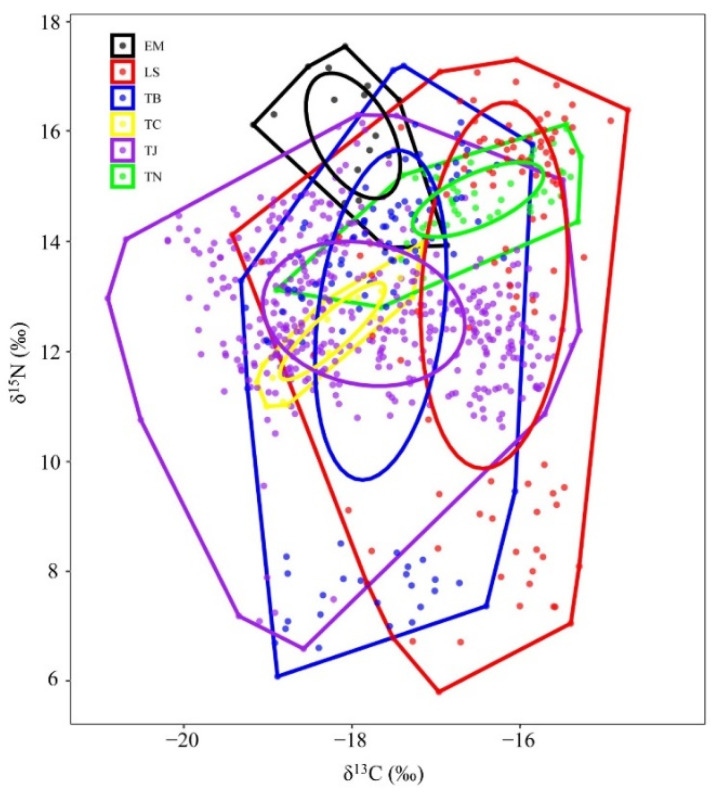
Two-dimension map of trophic niches of six species of Trichiuridae in the Chinese seas. TJ, *Trichiurus japonicus*; TN, *T. nanhaiensis*; TB, *T. brevis*; LS, *Lepturacanthus savala*; EM, *Eupleurogrammus muticus*; TC, *Tentoriceps cristatus*.

The trophic niche widths of the six species of Trichiuridae could be classified into two groups: largehead hairtail, Chinese short-tailed hairtail, and Savalai hairtail were in the wide niche group, while South China Sea hairtail, smallhead hairtail, and crested hairtail were in the narrow niche group. There was significant niche overlap among species in the wide niche group, but no overlap occurred among species in the narrow niche group. Chinese short-tailed hairtail overlapped with the other five species in trophic niche, especially with largehead hairtail. Largehead hairtail also overlapped with Savalai hairtail and crested hairtail but remained distant from South China Sea hairtail and smallhead hairtail. No niche overlap appeared among South China Sea hairtail, smallhead hairtail, and crested hairtail (Figure 6).

The trophic structure parameters of the six hairtail species are shown in Table 3. The maximum values of CR and TA were found in largehead hairtail; those of NR, CD, and SDNND were found in Savalai hairtail; that of MNND was found in smallhead hairtail; and that of SEA was found in Chinese short-tailed hairtail. The minimum values of CR, NR, TA, and SEA were found in crested hairtail; those of MNND and SDNND were found in largehead hairtail; and that of CD was found in South China Sea hairtail.

## 4. Discussion

### 4.1. Composition and Distribution of Hairtails

Hairtail in the Chinese seas have been considered as one species named *Trichiurus haumela* [27]. In 1962, Zhu et al. [28] reported a new genus and species called *Pseudoxymetopon sinensis*, sampled in the deep-water area of the East China Sea, which was later proved to be a synonym of *Tentoriceps cristatus* [29]. Based on body shape and other morphological indicators, Lee et al. [30] distinguished hairtail in the Taiwan Strait into the two species of *Trichiurus japonicus* and *T. lepturus*. Although there has been much debate about the validity of *T. lepturus* ([31,32]), molecular evidence has confirmed the validity of both species and suggests that *T. lepturus* is the most recently evolved species, with a close relationship to *T. Japonicus* [33,34]. A new species, *Trichiurus nanhaiensis*, was later found at many sites, including the Beibu Gulf, Guangdong, Minnan-Taiwan shoal fishing ground, and the southeast Taiwan Strait [32,35]. In this study, six species of four genera of Trichiuridae were identified, all belonging to the whip-tailed type commonly appearing in coastal waters. Fork-tailed hairtail are mainly distributed in sea areas deeper than 100 m instead of common offshore economic fishing species [9].

Regarding the wide distribution and high production, the largehead hairtail is the dominant species of Trichiuridae in Chinese seas. Consistent with historical records [36,37], the distributional area of South China Sea hairtail was sampled only in the South China Sea, and Savalai hairtail showed a wide distribution covering the South China Sea and East China Sea, as well as some areas of the Bohai and Yellow seas. Chinese short-tailed hairtail have previously been recorded in the South China Sea [36,38]; however, in this study, this species was also collected in the East China Sea, indicating an expansion of distributional range. On the contrary, the distributional range of smallhead hairtail shrank from Chinese coastal waters [9] to only the two sites of Zhanjiang in the South China Sea and Lianyungang in the southern Yellow Sea. In addition, the distributional range of crested hairtail shrank from historical records in the East China Sea and the South China Sea [28,39] to local areas, such as Beihai and Shantou in the South China Sea.

### 4.2. Age and Growth

According to preanal length, the hairtail samples could be divided into different body size groups including large type (preanal length longer than 280 mm), medium type (preanal length 210–280 mm), and small type (preanal length shorter than 210 mm) [40]. In crested hairtail, most individuals were large; in largehead hairtail, most were from the medium and small types; and in smallhead hairtail, all individuals were of small type. In the remaining three species, more than 90% were from small type. In terms of weight division standards [40], the majority of largehead hairtail and South China Sea hairtail were medium (weight 125–200 g) and small individuals (weight lower than 125 g); in smallhead hairtail, all samples were of the small type. In the remaining three species, small individuals dominated the populations. All these factors indicated that the population of common species of Trichiuridae in Chinese seas showed a tendency toward miniaturization as a result of fishery-induced evolution [41]. Compared with historical data, the resource populations of common species of hairtail family in China’s coastal waters show a trend of miniaturization. Before the early 1980s, catches of hairtail were mainly medium-sized individuals, but they have been dominated by small individuals since the 1990s [40,42], which can be explained by excessive exploitation. Overfishing breaks the natural compensation mechanism of a population, leading to failure of effective population supplementation and resource decline in both production and individual miniaturization.

The values of allometric parameter *b* of the six common species of Trichiuridae in Chinese seas were all less than 3, showing negative allometric growth and indicating faster growth in length than in body height, which may be related to the flat and long body shapes of hairtail species. The relationship between body length and weight is not only affected by species physiological factors, but also by the environmental conditions of the sea and gender [43,44], e.g., the significant differences in growth between *T. leturus* populations in the Yellow and Bohai seas and the East China Sea [45]. The values of condition factor parameter *a*, an indicator reflecting habitat conditions [12], were quite different among the six species in this study, indicating environmental differences among the different sampling sites. The values of parameter *b* for species in northern Chinese seas were significantly lower than those of species inhabiting southern Chinese seas, and this fact could be attributed to differences in environmental and nutritional conditions [12,43]. Under economic development and climate change, the East China Sea is characterized by serious pollution, the expansion of hypoxia, and frequent algae blooms, so carnivorous hairtail in this area face a serious lack of food sources. In contrast, in the South China Sea, the high species diversity provides enough food for hairtails, so there was little niche overlap among the hairtail species distributed in this area.

The common species of Trichiuridae in Chinese seas in this study were mainly 1–2 years of age, accounting for more than 80% of the total samples; especially in South China Sea hairtail, Chinese short-tailed hairtail, and Savalai hairtail, no individuals older than 3 years of age were sampled. In the 1960s and 1980s, the age range of largehead hairtail in the East China Sea was 1–8 years, declining to 1–4 years in the 1990s, with the dominant age range 1–2 years [40]. In the 1990s, the age range of South China Sea hairtail in the Taiwan Strait was 1–7 years, mainly in the range of 3–4 years (75%) [46]. The age compositions of both the largehead hairtail and South China Sea hairtail populations have changed significantly, characterized by obvious sexual precocity and individual miniaturization. There are no previous reports on the ages and growth of Savalai hairtail and Chinese short-tailed hairtail; however, according to the data in this study, the two species were dominated by younger individuals mostly less than 1 year of age, suggesting that the population is younger.

### 4.3. Trophic Characteristics

The carbon stable isotope values of the six species of Trichiuridae in Chinese seas were similar with high overlaps in ranges and averages, suggesting mutual predation among the species [47,48,49], as well as obvious food competition. Largehead hairtail, Savalai hairtail, and Chinese short-tailed hairtail showed wide ranges of food sources, giving strong adaptability against food scarcity, while crested hairtail with narrow food choices were sensitive to deterioration of food conditions. The trophic levels of marine organisms along Chinese coastal waters generally varied within 0.0–4.5 [50,51]. The six species of Trichiuridae in this study occupied the top of the food web, with trophic levels higher than 3. As a carnivorous species with migratory habits, hairtails played a bottom-up role in regulating the trophic dynamics of marine food webs.

The TA, representing the total area of niche space, of largehead hairtail was the largest, while the MNND and SDNND, representing trophic similarity, were the smallest among the six species, which suggested that largehead hairtail showed the highest stability and the greatest impact on the stability of the trophic structure. Savalai hairtail showed a few overlaps with other species in SEA and deviation in CR, helping the species avoid competition. Although largehead hairtail and Chinese short-tailed hairtail overlapped in SEA, the expansion of CR in the former species and the expansion of NR in the latter species indicated that the two species reduced competition for coexistence by diversifying food sources and trophic levels, respectively. Species in the South China Sea showed narrow distributional range and limited biomass, leading to little competition. However, these species are weak in food utilization, poor in environmental adaptation, and weak in competition [48,52], making it difficult to survive under the pressure of intense fishing and climate change.

In Chinese seas, largehead hairtail and Savalai hairtail were the two most important populations that constituted catch production, followed by Chinese short-tailed hairtail and smallhead hairtail. South China Sea hairtail and crested hairtail were seldomly sampled, representing by-catch populations in production. Benefitting from species traits, such as strongest migratory ability, a wide range of food selectivity, and the evolutionary trends of sexual precocity and individual miniaturization, largehead hairtail successfully maintained high production [4], even under overfishing and environmental degradation. However, the effective recovery and reasonable utilization of hairtail resource are still the focuses of attention for Chinese marine fishery.

## 5. Conclusions

The results of the present study showed that six species of four genera and two subfamilies of Trichiuridae were identified, and largehead hairtail and Savalai hairtail were the two most productive species. Species mostly inhabiting the northern parts of Chinese seas showed higher values of growth parameter *b* than those in southern parts. Under multiple stresses, hairtails showed obvious sexual precocity and individual miniaturization. Hairtails occupied the top of the food web and played a bottom-up role in regulating the trophic dynamics of marine food webs. Largehead hairtail showed the highest stability and the greatest impact on the stability of the trophic structure. The effective recovery and reasonable utilization of hairtail resources are still the primary focuses of attention for Chinese marine fishery.

## Figures and Tables

**Table 1:** Geographical information of fishing sites.

Sea Area	Site Number	Site Name	Longitude	Latitude
Bohai and Yellow Seas	1	Dandong (DD)	123.90	40.00
	2	Dalian (DL)	121.50	39.00
	3	Weihai (WH)	122.20	37.50
	4	Qingdao (QD)	120.20	36.00
East China Sea	5	Lianyungang (LY)	119.40	34.70
	6	Nantong (NT)	121.00	32.00
	7	Zhoushan (ZS)	122.50	30.20
	8	Wenzhou (WZ)	121.00	28.00
	9	Ningde (ND)	120.20	27.00
	10	Quanzhou (QZ)	118.70	24.80
South China Sea	11	Shantou (ST)	116.50	23.25
	12	Shenzhen (SZ)	114.00	22.50
	13	Yangjiang (YJ)	111.91	21.63
	14	Zhanjiang (ZJ)	110.50	21.00
	15	Beihai (BH)	109.20	21.60
	16	Wenchang (WC)	110.75	19.60
	17	Sanya (SY)	109.60	18.30

**Table 2:** Preanal length and weight of six species of Trichiuridae in the coastal waters of China.

Species	Preanal Length (mm)			Weight (g)		
	Min	Max	Mean ± SD	Min	Max	Mean ± SD
*Trichiurus japonicus*	150	517	230 ± 44	38.9	1731.7	192.2 ± 143
*Trichiurus nanhaiensis*	143	382	195 ± 40	46.8	787.3	123.0 ± 114
*Trichiurus brevis*	127	236	177 ± 21	34.5	211.6	98.0 ± 35
*Lepturacanthus savala*	79	239	183 ± 23	12.8	206.6	99.5 ± 32
*Eupleurogrammus muticus*	87	142	113 ± 11	10.5	37.4	22.4 ± 6
*Tentoriceps cristatus*	215	321	264 ± 25	51.6	204.2	113.5 ± 39

**Table 3:** Parameters of trophic structure of six hairtail species CR, range of δ^13^C; NR, range of δ^15^N; TA, total area of the convex hull; CD, mean distance to centroid; MNND, mean nearest neighbor distance; SDNND, standard deviation of nearest neighbor distance; SEA, standard ellipse area.

Species	CR	NR	TA	CD	MNND	SDNND	SEA
*Trichiurus japonicus*	5.61	9.71	35.77	1.57	0.12	0.13	4.80
*Trichiurus nanhaiensis*	3.62	3.32	6.04	0.85	0.19	0.21	1.22
*Trichiurus brevis*	3.47	11.11	28.67	2.58	0.22	0.21	6.90
*Lepturacanthus savala*	4.70	11.50	35.34	3.01	0.28	0.22	8.82
*Eupleurogrammus muticus*	2.30	3.66	4.37	1.07	0.33	0.17	1.69
*Tentoriceps cristatus*	1.98	2.98	2.07	0.92	0.23	0.14	0.80

**Figure A1:**
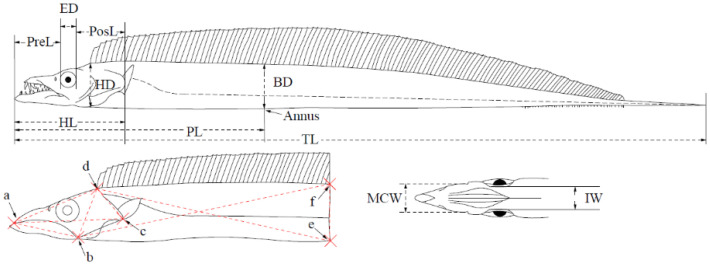
Measurements of morphological characteristics of hairtail for species identification. ED: Eye diameter; PreL: Preorbital length; PosL: Postorbital length; HL: Head length; PL: Preanal length; TL: Total length; HD: Head depth; BD: Body depth; IW: Interorbital width; MCL(ab): Mouth crack length; MCW: mouth crack width; ac; ad; bc; bd; be; bf; dc; de; df.

**Figure A2:**
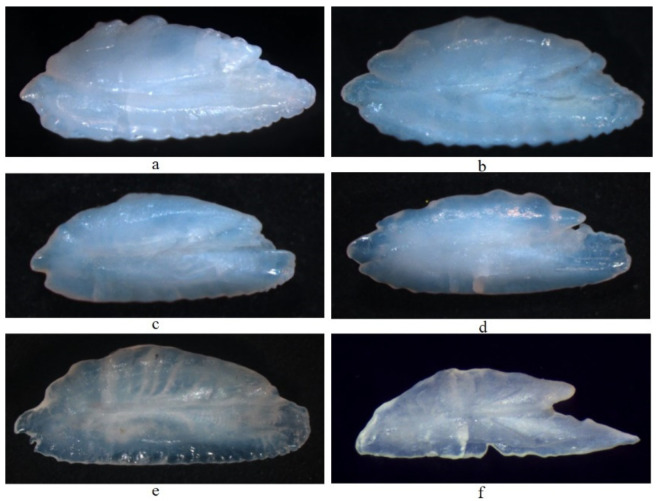
Morphology of otolith of six hairtail species. Note: (**a**), *T. japanicus*; (**b**), *T. nanhaiensis*; (**c**), *T. brevis*; (**d**), *L. savala*; (**e**), *E. muticus*; (**f**), *Te. Cristatus*.

**Figure A3:**
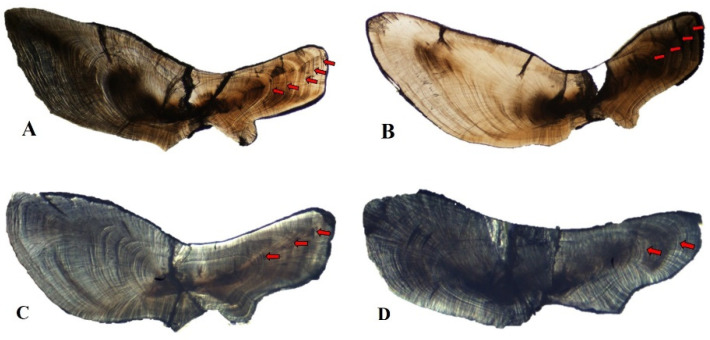
Age determination from otolith of largehead hairtail *Trichiurus japonicus*. (**A**), 6 years; (**B**), 5 years; (**C**), 4 years; (**D**), 3 years.

**Figure A4:**
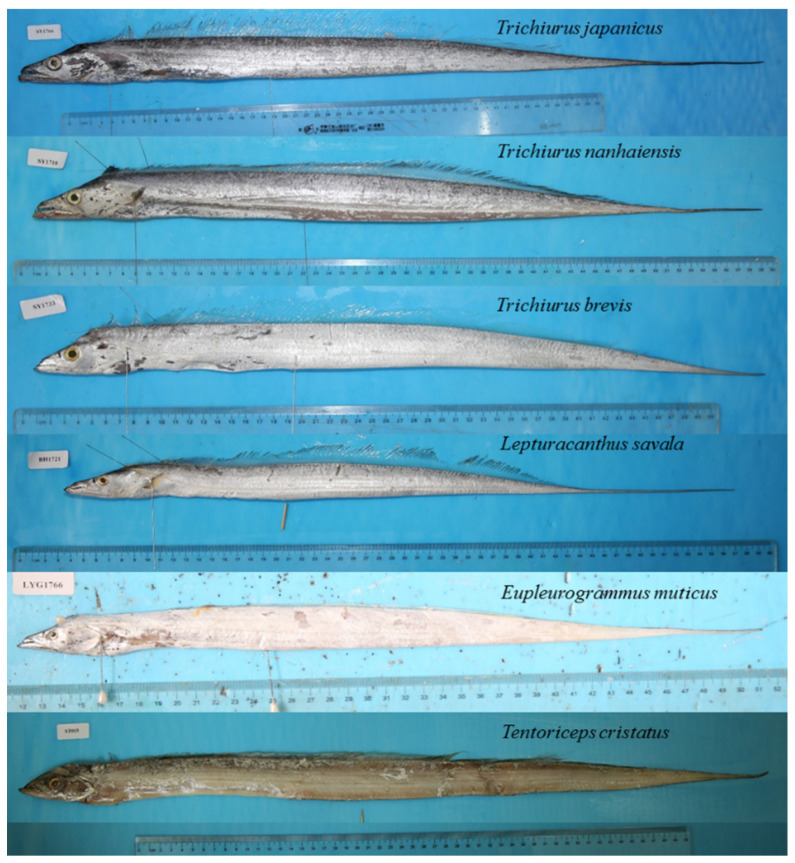
Photos of six species of Trichiuridae in the coastal waters of China.

**Figure A5:**
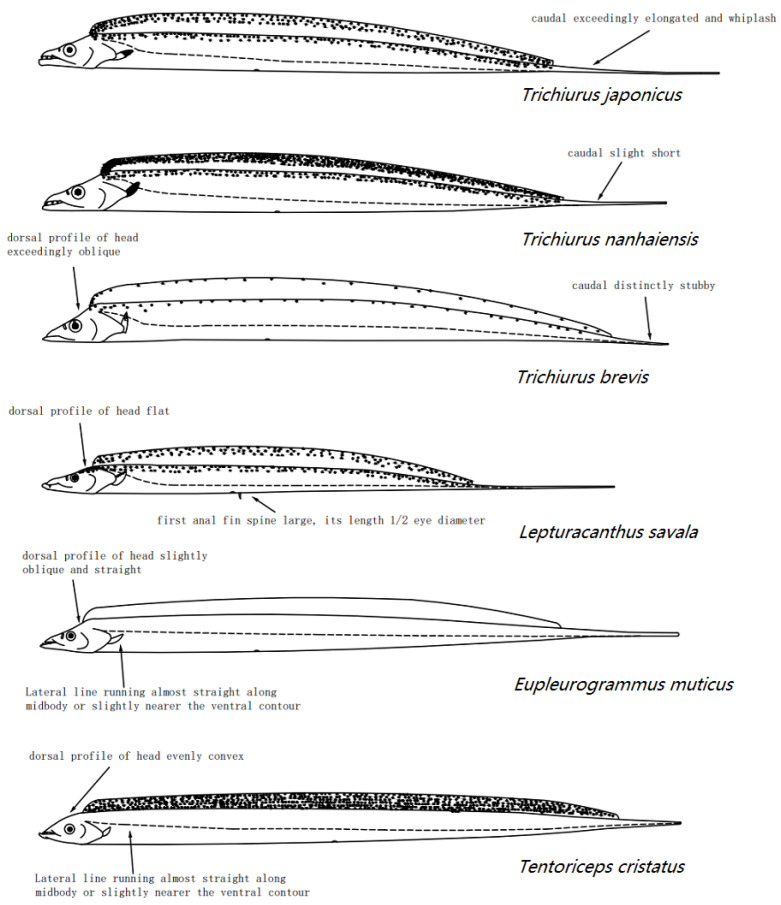
Hand drawing of six hairtails in the Chinese seas, with details of morphological characteristics for species identification.

## Data Availability

To obtain data from this study, please contact author Xiongbo He (email: xiongbo@163.com).

## Appendix B

The sagittal otoliths of the six species of hairtail were similar in shape, similar to grains of rice. The shape of the adaxial plane was narrow and long oval. The dorsal and ventral sides of the otoliths showed wavy protrusions or serrated leaf shaped crystal protrusions, and the ventral side was smoother than the dorsal side. The otolith was provided with a main interproximal groove; the main interproximal groove extended from the front end to the rear end of the otolith; the groove in the front end was obvious, and became shallower with backward extension. The front end of the otolith contained a base leaf and a wing leaf: the base leaf was longer than the wing leaf, sharp in the front end. There was no base leaf at the back end of the otolith but a leaf-like protrusion. The longitudinal section of otolith grinding was observed under the optical microscope. The grinding section of otolith was nearly arc-shaped, with center at the intersection of the middle depression of the arc, and outward radiating cracks from the center. A regular alternating growth zone of transparent broad band and dark narrow band was formed around the center of otolith to the edge of otolith, which was regarded as the basis for age determination.

## Appendix C

Identification key for six species of Trichiuridae taxonomy:

- 1(2) Lacrymal bone large; two-thirds of premaxilla sheathed by lacrymal bone. Black blotch in pectoral fin base present. Pelvic girdle and fin present, but fin reduced to a scale-like process. Dorsal soft rays 96~134, anal soft rays 73~106, pectoral soft rays 8~11, gill rakers 5~12 + 2~9……………………………………*Eupleurogrammus muticus* (Gray, 1831).
- 2(1) Lacrymal bone small; only one-third of premaxilla sheathed by lacrymal bone. Black blotch in pectoral fin base absent.
- 3(4) Pectoral fins short, not reaching lateral line. Dorsal profile of head evenly convex. Dorsal soft rays 129~145, anal soft rays 71~93, pectoral soft rays 10~11, gill rakers 8~11 + 4~5……………………………………………….*Tentoriceps cristatus* (Klunzinger, 1884).
- 4(3) Pectoral fins extending beyond lateral line. Dorsal profile of head flat.
- 5(6) First anal fin spine large, with length one-half of eye diameter; soft anal fin ray pungent spinules breaking through ventral skin. Pectoral fins yellow. Iridescence of eyes dose not turn yellow after death. Dorsal soft rays 91~134, anal soft rays 64~97, pectoral soft rays 10~11, gill rakers 0~8 + 0~45………………………………*Lepturacanthus savala* (Cuvier, 1829).
- 6(5) First anal fin spine small, with length less than pupil diameter; soft anal fin rays slightly breaking through ventral skin in smaller specimens. Pectoral fins grey or white.
- 7(8) Preanal length more than three times total length. Dorsal fin silver with transparency. Eye iridescence not turning yellow after death. Dorsal soft rays 123~172, anal soft rays 73~142, pectoral soft rays 10~11, gill rakers 6~24 + 4~11……………………….…….……..*Trichiurus japanicus* Temminck & Schlegel, 1844.
- 8(7) Preanal length less than three times total length.
- 9(10) Iridescence of eyes turning yellow after death. Dorsal fin yellowish; pectoral fins grey. Osteoma in posterior of supraoccipital present. Dorsal soft rays 129~143, anal soft rays 83~110, pectoral soft rays 11, gill rakers 5~13 + 4~8…………………………………………..….*Trichiurus nanhaiensis* Wang & Xu, 1992.
- 10(9) Caudal distinctly stubby. Iridescence of eyes turning yellow after death. Dorsal fin silver. Osteoma in posterior of supraoccipital absent. Dorsal soft rays 106~140, anal soft rays 60~110, pectoral soft rays 10~11, gill rakers 4~11 + 2~9………………………………………………….*Trichiurus brevis* Wang & You, 1992.

**Table A1:** Morphological characteristics six hairtail fishes in the coastal waters of China.

Characteristics	TJ	TN	LS	TB	EM	TC
TL/PL	3.1	2.7	3.1	2.9	3.2	2.7
PL/HL	2. 8	2.6	2.6	2.7	3.0	3.6
HL/ED	6.9	6.8	9.6	6.4	7.3	6.3
HL/IW	6.8	7.4	6.5	6.9	8.6	8.6
HL/PreL	2.9	3.0	2.7	3.0	2.7	2.6
HL/PosL	2.0	1.9	1.9	2.0	2.0	2.2
BD/HD	1.1	1.1	1.1	1.1	1.2	1.0
HD/ED	3.2	3.6	4.2	3.3	3.5	3.0
HD/IW	3.2	3.9	2.8	3.6	4.1	4.1
PosL/PreL	1.5	1.6	1.4	1.5	1.4	1.2
MCL/MCW	4.0	4.5	4.7	4.3	4.0	5.8
Number of dorsal soft rays	123~172	129~143	91~134	106~140	96~134	129–145
Number of anal fin spine	73~142	83~110	64~97	60~110	73~106	71–93
Number of pectoral soft rays	10~12	11	10~11	10~13	8~11	10~11
Number of canine teeth of upper jaw	2~6	3~6	0~6	0~6	2~5	0–6
Number of canine teeth of lower jaw	0~3	1~2	0~3	0~3	0~2	0–3
Gill raker	6~24 + 4~11	5~13 + 4~8	0~8 + 0~4	4~11 + 2~9	5~12 + 2~9	8~11 + 4~5

Note: TJ: *Trichiurus japonicus*; TN: *T. nanhaiensis*; TB: *T. brevis*; LS: *Lepturacanthus savala*; EM: *Eupleurogrammus muticus*; TC: *Tentoriceps cristatus*; ED: Eye diameter; PreL: Preorbital length; PosL: Postorbital length; HL: Head length; PL: Preanal length; TL: Total length; HD: Head depth; BD: Body depth; IW: Interorbital width; MCL(ab): Mouth crack length; MCW: mouth crack width;.
